# Non-negative tensor factorization workflow for time series biomedical data

**DOI:** 10.1016/j.xpro.2023.102318

**Published:** 2023-07-07

**Authors:** Koki Tsuyuzaki, Naoki Yoshida, Tetsuo Ishikawa, Yuki Goshima, Eiryo Kawakami

**Affiliations:** 1 Laboratory for Bioinformatics Research, RIKEN Center for Biosystems Dynamics Research, Wako, Saitama 351-0198, Japan; 2 Japan Science and Technology Agency, PRESTO, 7 Gobancho, Chiyoda-ku, Tokyo 102-0076, Japan; 3 Department of Artificial Intelligence Medicine, Graduate School of Medicine, Chiba University, Chiba 260-8670, Japan; 4 Advanced Data Science Project (ADSP), RIKEN Information R&D and Strategy Headquarters, Yokohama, Kanagawa 230-0045, Japan; 5 Department of Extended Intelligence for Medicine, The Ishii-Ishibashi Laboratory, Keio University School of Medicine, Shinjuku-ku, Tokyo 160-8582, Japan; 6 NEXT-Ganken Program, Japanese Foundation for Cancer Research (JFCR), Koto Ward, Tokyo 135-8550, Japan; 7 Institute for Advanced Academic Research (IAAR), Chiba University, Chiba 260-8670, Japan

**Keywords:** Bioinformatics, Health Sciences, Computer sciences

## Abstract

Non-negative tensor factorization (NTF) enables the extraction of a small number of latent components from high-dimensional biomedical data. However, NTF requires many steps, which is a hurdle to implementation. Here, we provide a protocol for TensorLyCV, an easy to run and reproducible NTF analysis pipeline using Snakemake workflow management system and Docker container. Using vaccine adverse reaction data as an example, we describe steps for data processing, tensor decomposition, optimal rank parameter estimation, and visualization of factor matrices.

For complete details on the use and execution of this protocol, please refer to Kei Ikeda et al.^1^

## Highlights

• TensorLyCV framework to perform NTF
• Rank optimization using the masking approach to analyze high-dimensional biomedical data
• Snakemake and Docker workflow to analyze vaccine adverse reaction data

## Abstract (editor highlights)

Publisher’s note: Undertaking any experimental protocol requires adherence to local institutional guidelines for laboratory safety and ethics.

## Graphical abstract

**Figure:**
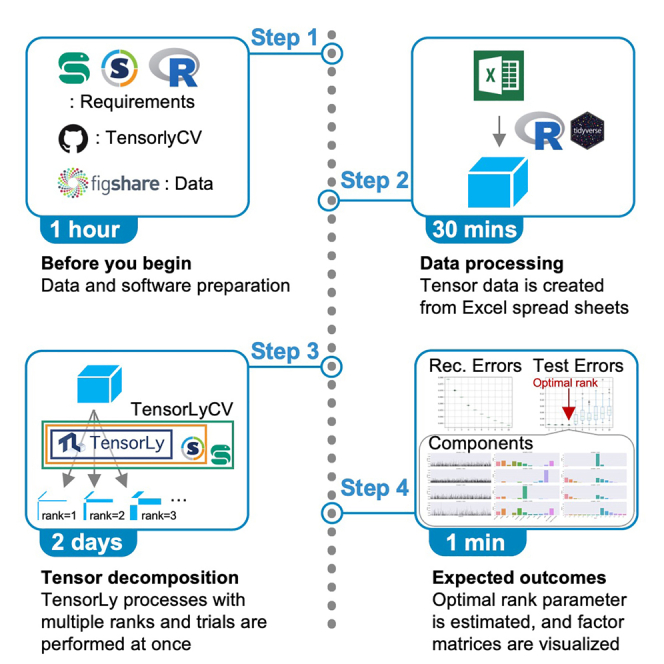

## Before you begin

This protocol describes a procedure for performing non-negative tensor factorization (NTF) on time-series biomedical data using a workflow called TensorLyCV. NTF requires multiple steps: software installation, data reshaping, rank estimation, decomposition at the optimal rank, and visualization of the tensor components. While the decomposition itself can be performed by existing Python^2^ and R^3^ packages such as TensorLy^4^ and nnTensor,^5^ the other processes require a combination of multiple packages, which is a hurdle for researchers unfamiliar with computational analysis. To make the NTF processes easily executable and reproducible, TensorLyCV uses Snakemake^6^ workflow management system and Docker^7^ container. TensorLyCV frees users from cumbersome software installation and version control, allowing them to reproducibly perform NTF procedures on their own data by changing only a few arguments.

NTF is a topic modeling approach that can decompose the dynamics of biomolecules, biological signals, and clinical symptoms into a small number of time-evolving components. By describing biological phenomena as an ensemble of components, associations with clinical outcomes can be efficiently examined and testable hypotheses about the underlying mechanisms can be generated. NTF has been applied to a variety of biomedical data with non-negative (≥ 0) values, including gene expression data, EEG data, fMRI data, and longitudinal EHR data.^8^^,^^9^^,^^10^^,^^11^^,^^12^^,^^13^^,^^14^ We recently applied NTF on time-series adverse reaction data after receiving the BNT162b2 mRNA COVID-19 vaccine.^1^ In this study, four time-evolving components were extracted to identify different component-specific associations with background factors and post-vaccination antibody titers.

One of the technical challenges in NTF is rank optimization. In unsupervised learning, such as NTF, the number of components (i.e., ranks) must be predetermined, but the number of potential components is often not obvious, especially for biomedical data. Traditionally, the elbow method^15^^,^^16^ is often used for rank optimization, but in practice, a sharp elbow may not exist, and the optimal rank cannot be unambiguously determined. Instead, we adopted a masking approach in Ikeda K. et al.’s study.^1^ In the masking approach, a certain percentage of the original data elements are masked as noise, and a rank that minimizes the error in the task of imputation of masked elements with NTF, is chosen as the optimal rank.. This approach is also called "denoising" or "unsupervised cross-validation (CV)" and the error of the masked elements is also called "test error" in the context of machine learning.^17^^,^^18^^,^^19^ TensorLyCV offers the option of rank optimization using a masking approach in addition to the elbow method.

Here, we present a comprehensive protocol for the NTF analysis using vaccine adverse reaction data from the recent study^1^ as an illustrative example and demonstrate the step-by-step data reshaping, data analysis, and visualization process.

### Computer system

To reproduce the analysis in Ikeda K. et al.'s study,^1^ here we use our original Snakemake workflow TensorLyCV (https://github.com/kokitsuyuzaki/TensorLyCV) to perform TensorLy,^4^ which is a Python^2^ package to execute tensor decomposition methods including NTF. This is because the most time-consuming part was the rank estimation with different rank parameters and initial values^1^ and therefore a computing environment that enables distributed computing such as GridEngine/Slurm is recommended. TensorLyCV is designed to work whether on a local machine such as a laptop or in a distributed computation environment just by switching the arguments. We have already confirmed that TensorLyCV properly worked on the four computers below. In the following sections, we will show the computational time on the Linux Server.

**Table:** 

Machine name	OS	CPU	Cores	RAM	Storage (type)
Linux Server	CentOS 7.0	Xeon E5-2670 v3 @ 2.30GHz	24	96 GB	2 TB (HDD)
Intel Mac Laptop	macOS Big Sur 11.6	Intel Xeon W @ 2.7 GHz	24	192 GB	2 TB (SSD)
M1 Mac Laptop	macOS Big Sur 11.5.2	Apple M1	8	16 GB	2 TB (SSD)
Windows Laptop with WSL2	Ubuntu 20.04.6 LTS on Windows 11 Pro	Intel (R) Core (TM)i7-105110U CPU @1.80GHz 2.30GHz	4	32 GB	951 GB (HDD)

### Software installation

**Timing: 1 h**

In this section, we describe how to install required software.

For preprocessing section later, download and install the latest R^3^ in CRAN (https://www.r-project.org).

***Note:*** The tools needed will vary depending on the user’s data. Here, we recommend the following R packages.

***Note:*** The following code line is in R language, to be inputted into R-console window.

> R -e "install.packages(c('readxl', 'writexl', 'tidyverse', 'einsum', 'abind', 'reticulate', 'rTensor'), repos='https://cloud.r-project.org', dependencies=TRUE)"

TensorLyCV is intended to be run by the snakemake command. Snakemake is a Python package. Therefore, after installing Python first, install Snakemake by using some Python package manager such as pip, conda, or mamba. To download and install Snakemake, follow the instructions on the installation page (https://snakemake.readthedocs.io/en/stable/getting_started/installation.html).

All necessary tools to perform TensorLyCV (e.g., TensorLy) have already been pre-installed in a Docker container (https://hub.docker.com/r/koki/tensorlycv_component). Snakemake uses Singularity^20^ to convert the Docker container image into a local executable file before using it for calculations. This allows users to perform their calculations in any computing environment (e.g., cluster machine) without being aware of their user privilege. To download and install Singularity, follow the instructions on the installation page (https://docs.sylabs.io/guides/3.0/user-guide/installation.html#).

Finally, download TensorLyCV and change the working directory. The following code line is in Bash script, to be inputted into terminal window.

> git clonehttps://github.com/kokitsuyuzaki/TensorLyCV.git.

> cd TensorLyCV

***Note:*** The binary files of Python, Singularity, and R to be downloaded and how to download them will vary depending on the machine's operation system (i.e., Windows, macOS, or Linux). To download the appropriate binary file, follow the instructions on each installation page above.

### Vaccine adverse reaction datasets

**Timing: 1min**

In this section, we describe how to download data for demonstration.

In TensorLyCV, the input data is assumed to be a binary file containing NumPy^22^ multi-dimensional array saved by numpy.save. The vaccine adverse reaction datasets can be downloaded below. The following code line is in Bash script, to be inputted into terminal window.

> wget --no-check-certificatehttps://figshare.com/ndownloader/files/38344040-O data/vaccine_tensor.npy

## Key resources table

**Table:** 

REAGENT or RESOURCE	SOURCE	IDENTIFIER
**Deposited data**		

Vaccine adverse reaction datasets	Ikeda K. et al., 2022^1^ This paper	https://figshare.com/ndownloader/files/38344040

**Software and algorithms**		

R (v4.2.1)	R Core Team, 2020^3^	https://www.r-project.org/
readxl (v1.4.1)	Wickham et al., 2019^22^	https://cran.r-project.org/web/packages/readxl
writexl (v1.4.1)	Wickham et al., 2019^22^	https://cran.r-project.org/web/packages/writexl
tidyverse (v1.3.2)	Wickham et al., 2019^22^	https://cran.r-project.org/web/packages/tidyverse
einsum (v0.1.0)	Ahlmann-Eltze, 2021^24^	https://cran.r-project.org/web/packages/einsuml
abind (v1.4.5)	Plate et al., 2016^23^	https://cran.r-project.org/web/packages/abind
reticulate (v1.26)	Ushey et al., 2022^25^	https://cran.r-project.org/web/packages/reticulate
rTensor (v1.4.8)	Li et al., 2018^26^	https://cran.r-project.org/web/packages/rTensor
TensorLyCV (v1.7.0)	This paper	https://github.com/kokitsuyuzaki/TensorLyCV
snakemake (v7.1.0)	Mölder et al., 2021^6^	https://snakemake.readthedocs.io
singularity (v3.8.0)	Kurtzer et al., 2017^20^	https://docs.sylabs.io/guides/3.0/user-guide
python (v3.11.0)	Van Rossum and Ddrake, 2009^2^	https://www.python.org/
numpy (v1.23.5)	Harris et al., 2020^21^	https://numpy.org
tensorly (v0.7.0)	Kossaifi et al., 2019^4^	http://tensorly.org/stable/index.html
pandas (v1.5.2)	The pandas development team, 2020^27^	https://pandas.pydata.org
matplotlib (v3.6.2)	Hunter, 2007^28^	https://matplotlib.org
seaborn (v0.12.1)	Waskom, M. L., 2021^29^	https://seaborn.pydata.org

## Step-by-step method details

### Data preprocessing

**Timing: 30 min**

In this section, we describe how to preprocess the demonstration data.

First, we show the procedure for constructing tensor data. A tensor can be considered a generalization of a matrix. For example, a third-order tensor is a three-dimensional array that stores values in the depth way in addition to the vertical and horizontal ways. When saving such high-dimensional data (e.g., 3D) as a 2D Excel spreadsheet, several data types can be considered. Here, we introduce the procedures for constructing tensor data from three data types. In subsequent demonstrations, we will use only a portion of the data taken from Ikeda K. et al.^1^ The following code lines in this section are in R language, to be inputted into R-console window.

1. Start R and load the following packages:

> library("readxl")

> library("writexl")

> library("tidyverse")

> library("einsum")

> library("abind")

> library("reticulate")

> library("rTensor")

2. To set up the subsequent analysis, type as follows:

> metadata_name <- c("ID", "Sex", "AgeCategory", "BMICategory", "ShotSite", "ShotInterval", "IntervalToTest", "PostTiterLog2", "Antiinflammatory")

> symptoms <- c("joint_pain", "fatigue", "fever", "cold",

 "headache", "muscle_pain", "nausea", "flare",

 "swelling", "pain", "use_painkiller", "medical_checkup")

> days <- c(paste(1, 1:7, sep="_"), paste(2, 1:7, sep="_"))

> thr <- length(symptoms) ∗ length(days) ∗ 0.3

3. Converting 2D data to 3D.

**Figure 1:**
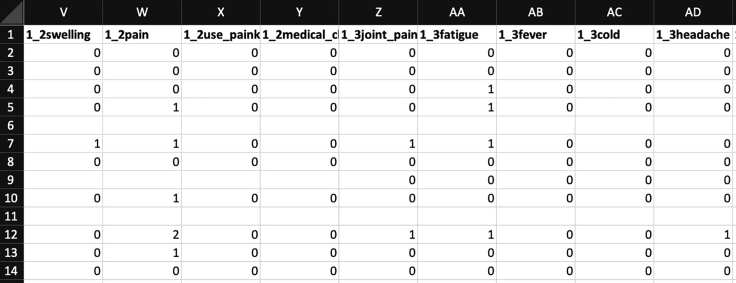
Type 1: Wide short data Rows indicate subjects and columns indicate symptoms and days.

**Type 1: Wide short data:** The first type is wide short matrix data, with rows for subjects and columns for combinations of days and symptoms (https://figshare.com/ndownloader/files/38365868, Figure 1). To load this Excel file in R, use read_excel^22^ function as follows:

> read_excel("type1_wide.xlsx") -> data1

Then, a set of matrices stratified by days is created with the map^22^ function, and they are stacked in the depth direction with the abind^23^ function to construct a third-order tensor called vaccine_tensor.

> map(days, function(x){data1 %>% select(contains(paste0(x, symptoms)))}) %>% abind(along=3) -> vaccine_tensor

**Figure 2:**
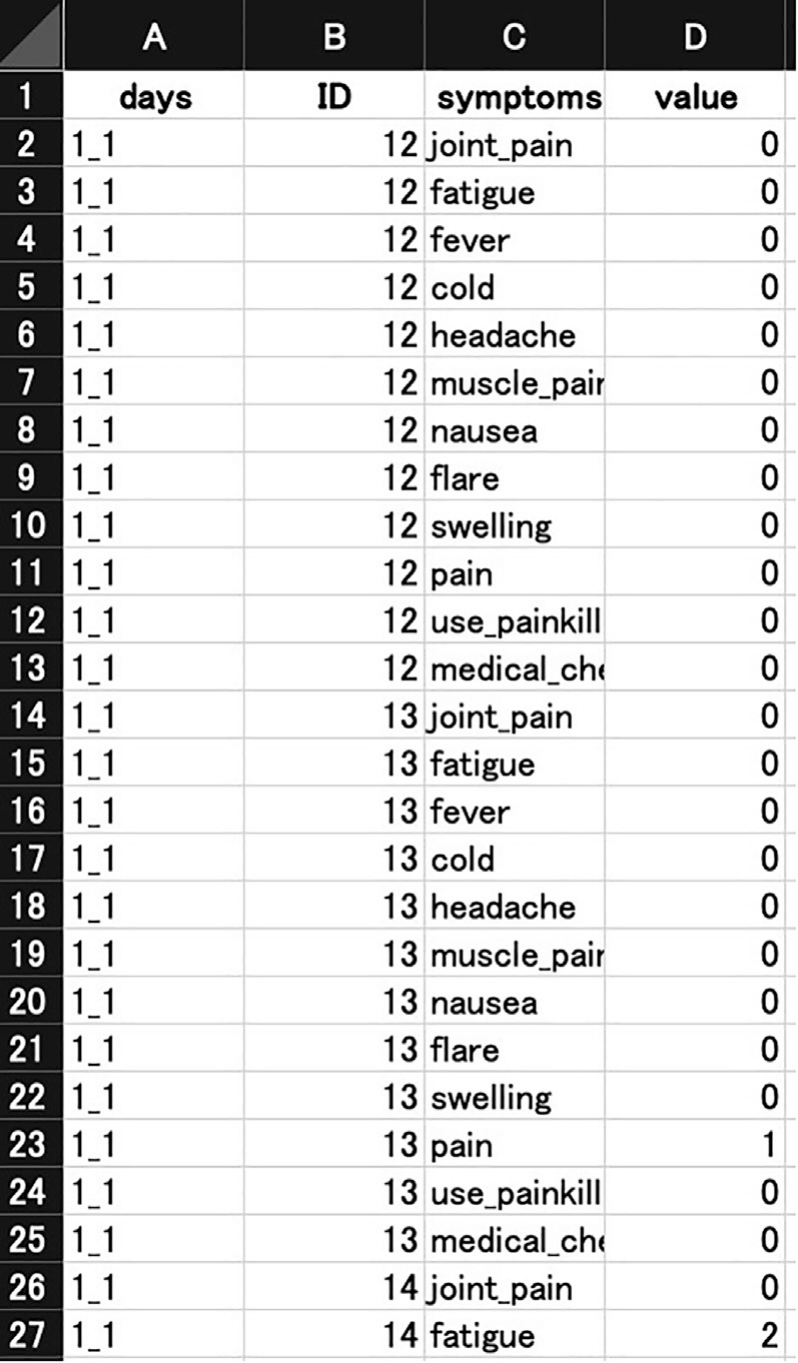
Type 2: Tall narrow data (also known as "tidy" data) Rows indicate subjects and columns indicate all the variables corresponding to each subject.

**Type 2: Tall narrow data:** The second type is tall narrow data also known as "tidy" data^22^ (https://figshare.com/ndownloader/files/38362235 Figure 2). To load this Excel file in R, use read_excel function as follows:

> read_excel("type2_long.xlsx") -> data2

Then, a tidy dataset stratified by days is created with the map function, reshaped as a matrix form by pivot_wider^22^ and as.matrix, and they are stacked in the depth direction with the abind function to construct a third-order tensor called vaccine_tensor.

> subjects <- as.character(sort(unique(data2$ID)))

template <- matrix(NA, nrow=length(subjects), ncol=length(symptoms))

> dimnames(template) <- list(subjects, symptoms)

> map(days, function(x){data2 %>% filter(., days==x) %>% pivot_wider(., names_from="symptoms") -> tmp

 template[as.character(tmp$ID), symptoms] <- as.matrix(tmp[, symptoms])

 template

 }) %>% abind(along=3) -> vaccine_tensor

**Figure 3:**
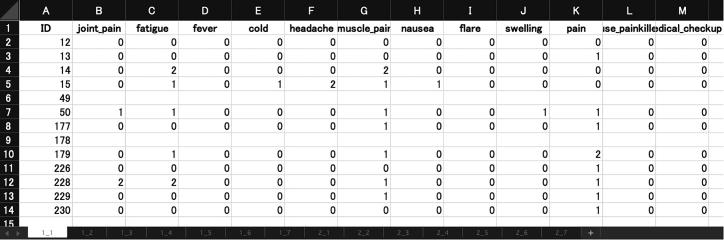
Type 3: Multiple sheets data After stratifying Type 1 data by days, multiple matrices are saved in each sheet.

**Type 3: Multiple sheets data:** The third type is one in which Type 1 data is pre-stratified by days and stored as separate sheets (https://figshare.com/ndownloader/files/38362238, Figure 3). This format may be the most straightforward format to create tensor data; this can omit the stratification part in the procedure for Type 1 data above and each sheet is directly stored as a frontal slice of vaccine_tensor as follows:

> map(seq_along(days), function(x){

 read_excel("type3_multisheets.xlsx", sheet=x, range=cell_cols("B:M"))

}) %>% abind(along=3) -> vaccine_tensor

4. Filter low-quality data.

***Note:*** In Ikeda, K. et al.'s study,^1^ we added the process for filtering the subjects with few observations (i.e., subjects whose observed elements are under 30%). This process is the summation of a 3D tensor for a given dimension and transforming it to 1D and can be easily described by using the einsum^24^ function, which is inspired by Einstein's summation.

> vaccine_tensor %>% is.na %>% einsum('ijk->i', .) %>% `<`(thr) %>% which -> subjects

5. Save tensor data as a NumPy binary file.

> np <- import("numpy")

> np$save("demo_data.npy", r_to_py(vaccine_tensor))

6. Finally, convert the vaccine_tensor to NumPy’s binary file.

***Note:*** This conversion can be performed by reticulate^25^ package. The third-order tensor data (1516 subjects × 12 symptoms × 14 days) for all subjects can be obtained from Figshare (see key resources table) and be used in the next section.

### Non-negative tensor factorization

**Timing: two days**

In this section, we describe how to perform TensorLyCV.

**Figure 4:**
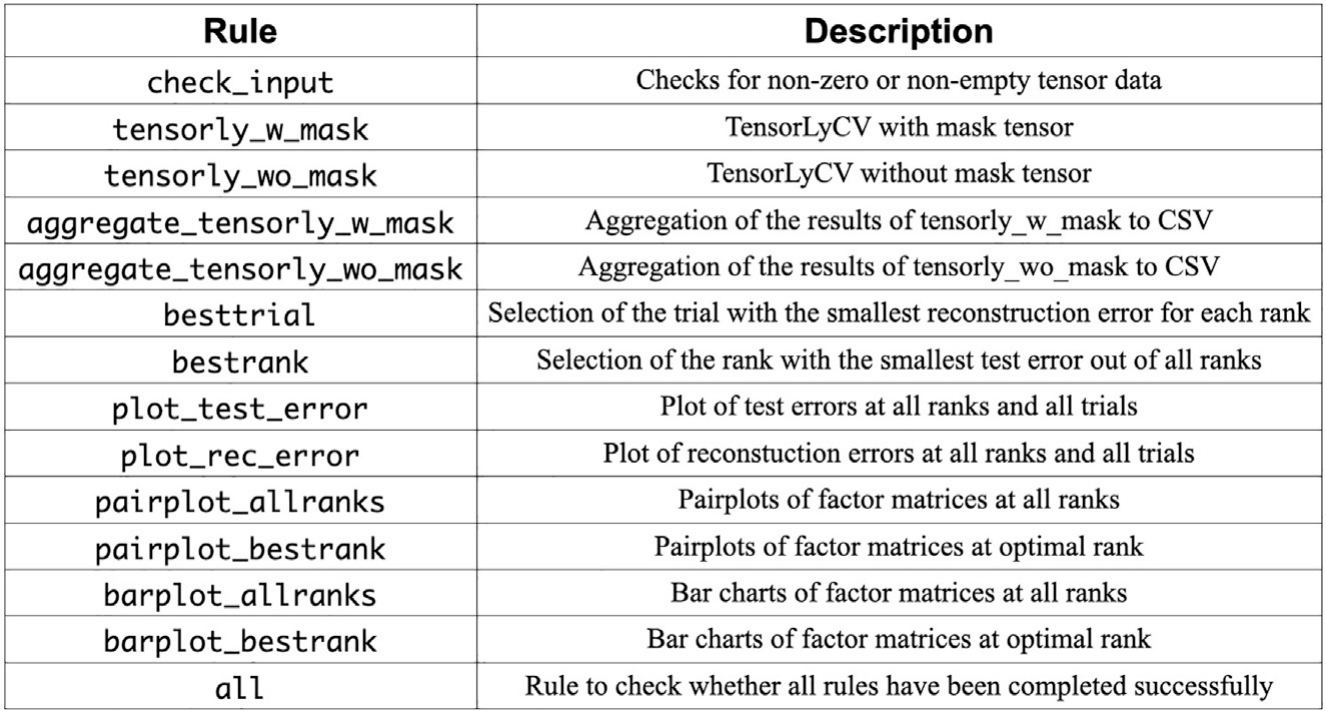
Rules and the descriptions in TensorLyCV

**Figure 5:**
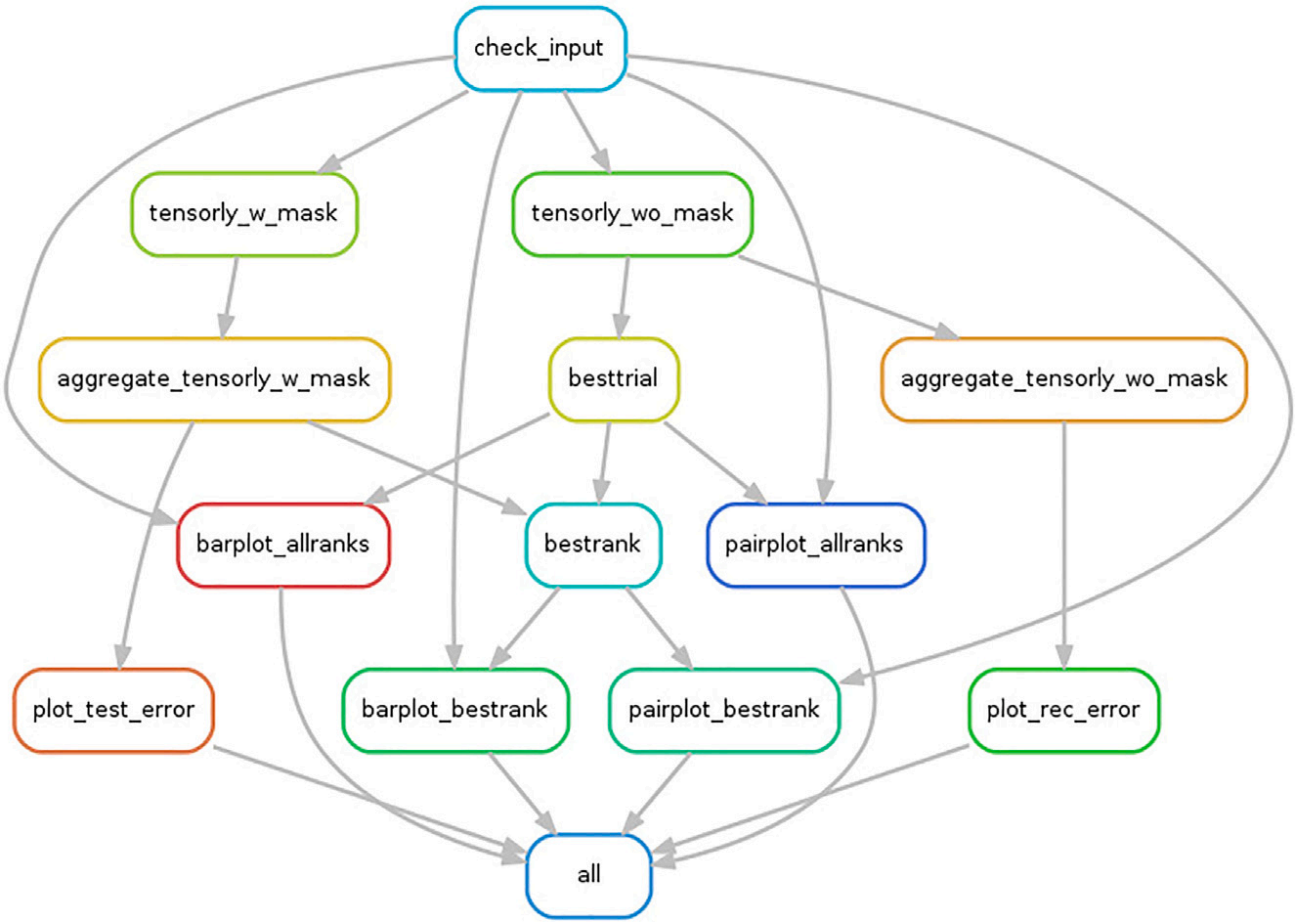
Dependency graph between rules in TensorLyCV If a rule on the graph is successfully executed, its downstream rule is executed. This figure is generated by Snakemake’s --rulegraph option (cf. https://github.com/kokitsuyuzaki/TensorLyCV/blob/main/src/dag.sh).

**Figure 6:**
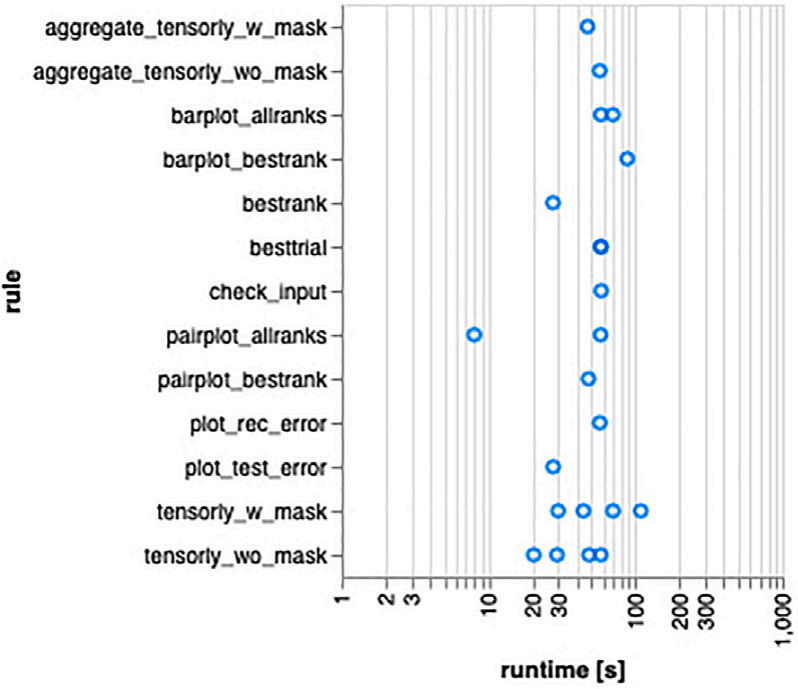
Computational time of each rule in TensorLyCV The x-axis indicates the calculation time of each rule and the y-axis indicates rules in TensorLyCV. This figure is generated by Snakemake’s --report option (cf. https://github.com/kokitsuyuzaki/TensorLyCV/blob/main/src/report.sh).

TensorLyCV consists of 14 rules, and once a rule is successfully executed, the downstream rule is then executed, and this procedure is repeated to ensure that all calculations are properly finished (Figures 4 and 5). The most time-consuming one of these rules is "tensorly_w_mask", which performs the rank estimation with masking approach (Figure 6). To accelerate this step, user has some options to perform TensorLyCV like below. The following code lines in this section are in Bash script, to be inputted into terminal window.

1. Execute the following Snakemake workflow.

On a local machine such as a laptop, run TensorLyCV as follows:

> snakemake -j 5 --config input=data/vaccine_tensor.npy outdir=output rank_min=1 rank_max=10 trials=50 n_iter_max=1000 ratio=30 --resources mem_gb=10 --use-singularity

**CRITICAL:** The above is a code that performs a series of NTF analyses at once, including rank estimation using masking approach, decomposition at the optimal rank, and visualization of the decomposition results. The argument -j is the number of CPU cores to be used in Snakemake. The arguments input and outdir are the input file and output directory, respectively. The arguments associated with rank estimation are rank_min, rank_max, trials, and n_iter_max, meaning a decomposition from rank 1 to 10 with 50 random trials using different initial values for each rank, up to 1,000 iterations before convergence on each random trial, and ratio is an argument related to the masking approach, meaning the percentage of elements to be masked as noise. Previous research^1^ has shown that varying the percentage of noise by 5%, 10%, 20%, and 30% does not affect the rank estimation, but noise above 40% makes the decomposition unstable and should be avoided. The argument mem_gb is the memory usage. --use-singularity is the argument to use Docker container via Singularity.

On a distributed environment with GridEngine, run TensorLyCV as follows:

> snakemake -j 32 --config input=data/vaccine_tensor.npy outdir=output rank_min=1 rank_max=10 trials=50 n_iter_max=1000 ratio=30 --resources mem_gb=10 --use-singularity --cluster "qsub -l nc=4 -p -50 -r yes"

**CRITICAL:** Here, the argument --cluster is added to use a distributed environment, and the command "qsub -l nc = 4 -p -50 -r yes" is given when submitting jobs to GridEngine.

On a distributed environment with Slurm, run TensorLyCV as follows:

> snakemake -j 32 --config input=data/vaccine_tensor.npy outdir=output rank_min=1 rank_max=10 trials=50 n_iter_max=1000 ratio=30 --resources mem_gb=10 --use-singularity --cluster "sbatch -n 4 --nice=50 --requeue"

**CRITICAL:** The arguments other than --cluster are the same as for GridEngine, but the command enclosed in "" after -cluster has been changed for Slurm.

Because TensorLyCV itself is also dockerized, if Docker is available, user can perform TensorLyCV by docker command as follows:

> docker run --rm -v $(pwd):/work ghcr.io/kokitsuyuzaki/tensorlycv:main -i /work/data/vaccine_tensor.npy -o /work/output --cores=5 –-rank_min=1 --rank_max=10 --trials=50 --n_iter_max=1000 --ratio=30 --memgb=100

**CRITICAL:** In this case, the installation of Snakemake and Singularity can be omitted.

***Optional:*** If a user predetermines the optimal rank, either empirically or by using the elbow method, etc., the user can skip the rank estimation step and just decompose and visualize the decomposition results at the specified rank.

> snakemake -j 32 --config input=data/vaccine_tensor.npy outdir=output rank_min=4 rank_max=4 trials=50 n_iter_max=1000 ratio=30 --resources mem_gb=10 --use-singularity

**CRITICAL:** By setting rank_min and rank_max to the same value (e.g., 4), no rank estimation is performed, and the decomposition is directly performed at the specified rank.

## Expected outcomes

**Figure 7:**
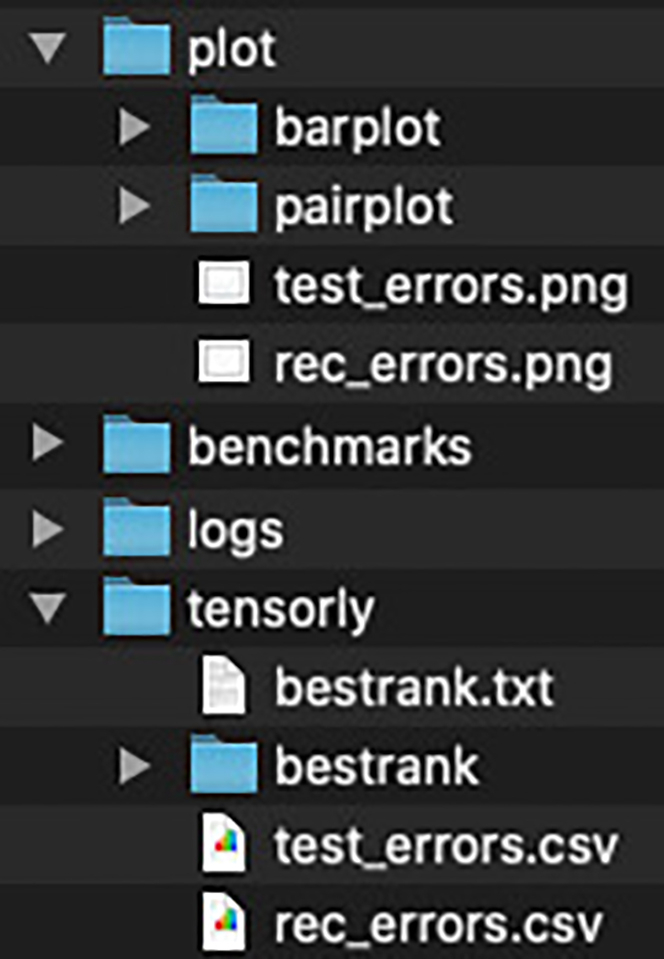
Output directories of TensorLyCV

If TensorLyCV runs successfully, many files and directories are generated in the directory specified by the outdir argument (e.g., *output*, Figure 7). The directory *tensorly* contains all the results of TensorLy with different ranks and initial values. The directory *plot* contains only the images of the trials with the smallest reconstruction error in all ranks. The directory *benchmarks* contains the calculation time and memory usage of all the processes in TensorLyCV and are used to generate the report (Figure 6). The directory *logs* contains the log files of all the processes in TensorLyCV and are referenced during debugging if the calculation fails for some reason.

**Figure 8:**
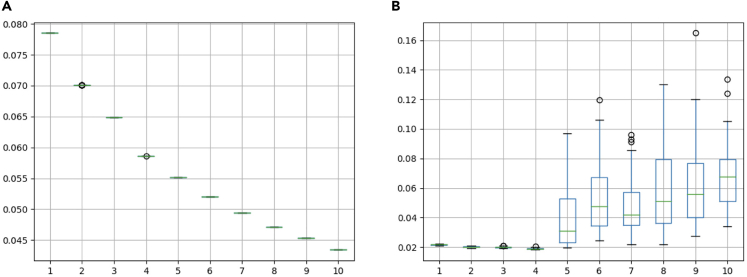
Reconstruction errors of NTF Reconstruction errors calculated as the mean squared error (MSE) for all elements without mask (A) and using the masking approach (B). The x-axis indicates rank parameters and the y-axis indicates the reconstruction errors.

*rec_errors.png* in the *plot* directory (Figure 8A) shows the reconstruction error calculated as the mean squared error (MSE) for all elements without mask and *test_errors.png* (Figure 8B) shows the reconstruction error using the masking approach (CV). These plots are generated from *tensorly/rec_errors.csv* and *tensorly/test_errors.csv* (Figure 7) by the rules plot_test_error and plot_rec_error (Figures 4 and 5). The small reconstruction error means that the original data is accurately represented by the NTF components. The boxplot shows the distribution of the reconstruction error obtained by 50 independent decomposition trials. Conventionally, the elbow method is applied to the curve in Figure 8A to find the optimal rank, but as can be seen from the figure, the decrease is gradual, and it is difficult to determine a clear elbow. Instead, TensorLyCV is designed to select the rank with the lowest median in Figure 8B, where rank = 4 is chosen as the optimal rank.

**Figure 9:**
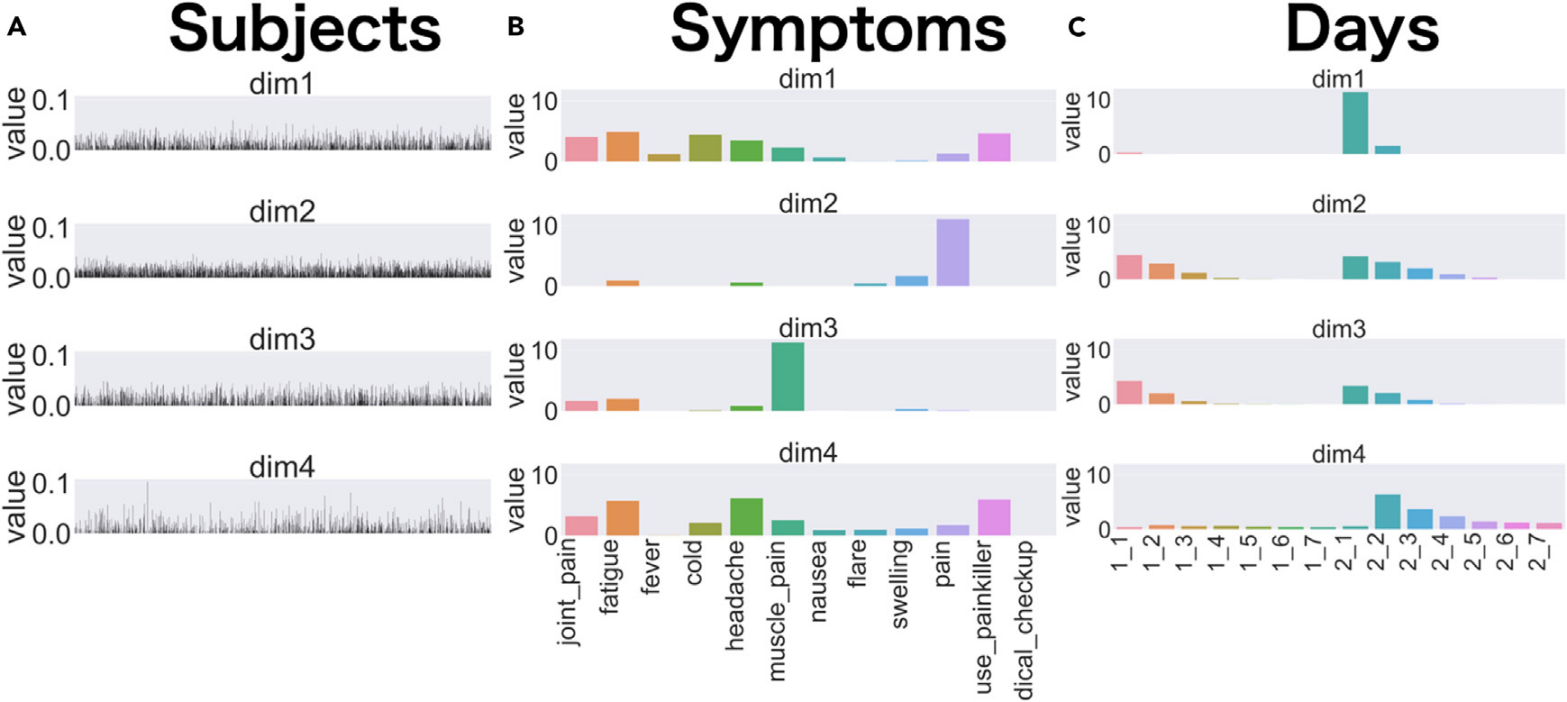
Tensor components identified with NTF Since the original vaccine adverse reaction data is a tensor consisting of three axes: subject, symptom, and time, the subject module (A), symptom module (B), and time module (C) are obtained. Each component is represented by the outer product of the modules in a horizontal row.

In TensorLyCV, there are two types of decomposition: decomposition using mask tensor (tensorly_w_mask) and decomposition without mask tensor (tensorly_wo_mask, Figures 4 and 5). The former results are used for optimal rank estimation by the masking approach, while the latter results are used for decomposition at the optimal rank. The information related to the decomposition at the optimal rank is stored separately in various directories as a directory called *bestrank* such as *tensorly/bestrank*, *plot/barplot/bestrank*, and *plot/pairplot/bestrank*. In the directory *tensorly/bestrank*, the value of reconstruction error, factor matrices, and the result of TensorLy are stored as *error.txt*, *factor1.csv*, *factor2.csv*, *factor3.csv*, and *tensorly.pkl*. These files can be used for further downstream analysis such as non-linear dimensional reduction (e.g., t-SNE^30^ and UMAP^31^), clustering (k-means^32^^,^^33^ and spectral clustering^34^^,^^35^), and association analysis. In the directory *plot/barplot/bestrank*, *factor1.png*, *factor2.png*, and *factor3.png* are generated and these are barplot for each factor matrix at the optimal rank (Figure 9). Plots at all searched ranks are also available in the directories *plot/barplot/[1 to 10]*. Similar plots are also visualized as paired plots (i.e., *plot/pairplot/bestrank* and *plot/pairplot/[1 to 10]*).

Figure 9A shows the individual scores of the components. Higher scores indicate stronger symptoms for the individual. Here, the component in the second row has the highest score for most individuals. Figure 9B shows what kind of symptoms are included in the component: the symptoms in the first and fourth rows include a variety of systemic symptoms, while the second row component is composed almost exclusively of pain at the inoculation site and the third row component is composed of whole-body muscle pain. Figure 9C represents the timing of the components; the second and third row components show similar temporal changes, with the strongest on the first day after both the first and second inoculation, lasting about 3 days. On the other hand, the first row component is very strong only one day after the second inoculation, while the fourth row component appears mainly after the second inoculation as well, peaking two days after inoculation and persisting for seven days.

## Limitations

The bottleneck in TensorLyCV workflow is the masking approach (i.e., tensorly_w_mask). Assuming the same processing time for each TensorLy calculation, the order of computational time is O(RTI), where R is the number of rank parameters, T is the number of random trials with different initial values, and I is the number of iterations to converge the calculation. When we perform TensorLyCV against the vaccine adverse-reaction data,^1^ R is 10, T is 50, and I is 1,000, each computation time for the masking approach ranges from 1 to 5 min and the total calculation time was about 15 h. If a user’s data is larger than ours, the total calculation time will be larger. See the troubleshooting section for tips on speeding up TensorLyCV.

## Troubleshooting

### Problem 1

Unable to perform TensorLyCV on M1/M2 Mac (related to step-by-step method details, non-negative tensor factorization step).

### Potential solution

TensorLyCV is based on Singularity to use Docker containers (related to before you begin, software installation step), but Singularity is still unstable on M1/M2 Mac as of January 26, 2023. To be able to perform TensorLyCV on M1/M2 Mac, user has two options: installation of required tools by conda/mamba command manually and performing snakemake command without --use-singularity option, or using TensorLyCV by Docker. Detailed procedures are described below.

https://github.com/kokitsuyuzaki/TensorLyCV/blob/main/README_AppleSilicon.md.

### Problem 2

TensorLyCV crashed with the message "The data tensor contains negative elements..." (related to step-by-step method details, non-negative tensor factorization step).

### Potential solution

The tensor data has negative elements. NTD supposes that the input data has no negative elements. Therefore, in such a case, user must convert the tensor data into non-negative tensor data in some ways such as taking absolute values or translation to make the minimum value 0.

### Problem 3

TensorLyCV crashed with Python’s MemoryError (related to step-by-step method details, non-negative tensor factorization step).

### Potential solution

The size of the tensor data is too large, or the memory capacity of the machine is too small. In such a case, perform TensorLyCV on large memory computers with setting the large memory usage (e.g., --resources mem_gb = 500).

### Problem 4

TensorLyCV crashed with Python’s AssertionError (related to step-by-step method details, non-negative tensor factorization step).

### Potential solution

The arguments for TensorLyCV may not be properly provided to Snakemake. Check to see if there is any typo in the source-code you have executed.

### Problem 5

Some steps of TensorLyCV could be performed but it’s going to take an awful lot of time (related to step-by-step method details, non-negative tensor factorization step).

### Potential solution

The bottleneck in TensorLyCV workflow is the masking approach (i.e., tensorly_w_mask) and the order of computational time is O(RTI), where R is the number of rank parameters, T is the number of random trials with different initial values, and I is the number of iterations to converge the calculation (cf. Limitations). Therefore, reducing the difference between rank_min and rank_max, and setting small values for trials and n_iter_max will reduce the total computation time. If some distributed computation environment is available, user can perform TensorLyCV with grid schedular such as GridEngine and Slurm. Besides, Snakemake also supports cloud execution such as the Google cloud engine (https://snakemake.readthedocs.io/en/stable/executing/cloud.html). Switching the computational environment can be easily accomplished by adding some arguments to the snakemake command as described above.

### Problem 6

The estimated rank by TensorLyCV was too small (related to step-by-step method details, non-negative tensor factorization step).

### Potential solution

If the user can be convinced from prior knowledge of the data that rank 1 is unreasonably too small because the data contains multiple patterns, setting different values with TensorLyCV’s argument may improve the results. Empirically, if the ratio of masking elements is too large (e.g., 40%), it may focus only on global patterns in the data and overlook local patterns, hence the ranks may be underestimated. The same can also happen if the number of iterations is too small (e.g., 10). Therefore, setting a smaller ratio and a larger n_iter_max arguments may generate larger rank. Users can also determine the rank of decomposition themselves, empirically or otherwise. It should be noted, however, that there are few cases in biomedicine where the number of latent components is obvious, and there is arbitrariness involved in empirically determining rank.

## Resource availability

### Lead contact

Further information and requests for resources should be directed to and will be fulfilled by the lead contact, Koki Tsuyuzaki (koki.tsuyuzaki@gmail.com).

### Materials availability

This study did not generate new unique reagents.

## Data Availability

The raw vaccine adverse reaction datasets are available on Figshare and the source code used to perform the masking approach against TensorLy is publicly available on GitHub (see key resources table) and also archived on Zenodo: https://doi.org/10.5281/zenodo.7728198. Any additional information required to reanalyze the data reported in this paper is available from the lead contact upon request.
